# Characterization of two novel defensins Han-Def1 and Han-Def2 from *Hyalomma anatolicum*

**DOI:** 10.3389/fmicb.2026.1823759

**Published:** 2026-05-14

**Authors:** Tingxiang Luo, Zhen Ma, Qingyong Guo, Depeng Yang, Sen Yang, Wenxiu Hu, Qiya Wu, Zhengxiang Hu, Bayinchahan Gailike, Ercha Hu

**Affiliations:** 1 College of Veterinary Medicine, Xinjiang Agricultural University, Urumqi, Xinjiang Uygur Autonomous Region, China; 2 Xingjiang Key Laboratory of New Drug Research and Development for Herbivorous Animals, Urumqi, Xinjiang Uygur Autonomous Region, China; 3 Herbivorous Animal Biomedicine and Diagnostic Technology Engineering Research Center, Urumqi, Xinjiang Uygur Autonomous Region, China; 4 Xinjiang Regional Key Laboratory of Clinical Veterinary Medicine Research, Urumqi, Xinjiang Uygur Autonomous Region, China; 5 Urumqi Field Scientific Observation and Research Station for Animal Diseases, Ministry of Agriculture and Rural Affairs, Urumqi, Xinjiang Uygur Autonomous Region, China; 6 College of Grassland Science, Xinjiang Agricultural University, Urumqi, Xinjiang Uygur Autonomous Region, China; 7 Veterinary Medicine Postdoctoral Research Station of Xinjiang Agricultural University, Urumqi, Xinjiang Uygur Autonomous Region, China

**Keywords:** antibacterial activity, antioxidant activity, bioinformatics, defensins, hemolytic activity, *Hyalomma anatolicum*

## Abstract

**Background:**

*Hyalomma anatolicum* is an important vector of veterinary and zoonotic pathogens, posing potential threats to livestock production and public health. Against the background of rising antibiotic resistance worldwide, it is necessary to explore new antimicrobial candidates. Tick defensins exhibit stable structures and strong antimicrobial activity, making them a valuable resource for the development of novel antibacterial substances.

**Methods:**

This study identified and characterized two defensin genes, *Han-Def1 and Han-Def2*, from *H. anatolicum* that were upregulated upon bacterial challenge. The genes were cloned, recombinantly expressed, and assessed for antimicrobial, hemolytic, and antioxidant activities.

**Results:**

Bioinformatic analysis confirmed that Han-Def1 and Han-Def2 are cationic defensins with a conserved cysteine-stabilized *α*/*β* fold, clustering phylogenetically with other known ixodid defensins. Both recombinant proteins exhibited potent bactericidal activity against livestock-associated *Staphylococcus aureus*, *Enterococcus faecalis*, *Bacillus subtilis*, with Han-Def1 displaying superior efficacy. Notably, neither peptide inhibited the Gram-negative *Pseudomonas aeruginosa* and *Escherichia coli*. At a high concentration of 750 μg/mL (182 μM for rDef1 and 175 μM for rDef2), the hemolytic rate against sheep erythrocytes was <2%, indicating excellent biocompatibility. Both defensins also possessed *in vitro* antioxidant capacity.

**Conclusion:**

Collectively, Han-Def1 and Han-Def2 are low-toxicity, selective antimicrobial peptides with potential for developing therapeutics against drug-resistant Gram-positive infections in livestock.

## 1 Introduction

As important vectors of diverse pathogens, ticks are capable of transmitting a wide range of pathogenic microorganisms. According to incomplete statistics, approximately 10% of ticks worldwide harbor pathogens, including over 80 virus species and at least 52 bacterial species (comprising 20 *Rickettsia* species, 18 spirochete species, and 14 other bacterial pathogens) (Brites-Neto et al., 2015; Jongejan and Uilenberg, 2004). Notably, ticks are the second most important vector of zoonoses worldwide, only after mosquitoes (Brites-Neto et al., 2015). Specifically, Xinjiang has diverse terrain, and a variety of dominant tick species exist in different habitats (Chen et al., 2010). Among them, *Hyalomma anatolicum* is a medically important hard tick capable of transmitting multiple zoonotic pathogens. This tick species is mainly distributed in southern Russia, central Asia, Pakistan, India, Nepal, and eastern European countries (Serretiello et al., 2020); in China, it is primarily distributed in the northwest region, with populations present in Xinjiang (Yang et al., 2020). Its main parasitic hosts are domestic animals such as gerbils, cattle, horses, and sheep, with a small number also parasitizing wild animals (Madison-Antenucci et al., 2020). Additionally, this tick species has a strong ability to actively seek out hosts for parasitism, posing significant hazards to humans and domestic animals, which seriously threatens the development of animal husbandry as well as the health of humans and animals (Parola and Raoult, 2001).

For ticks, innate immunity is crucial for resisting microbial damage, and they maintain a strong immune defense capacity to limit the proliferation and transmission of these pathogens (Kopácek et al., 2010; Torina et al., 2020). During long-term adaptation, ticks have developed an efficient innate immune system, with coordinated innate immune mechanisms that help defend against pathogen invasion (Fogaça et al., 2021; Sonenshine and Macaluso, 2017). When tick immune cells sense pathogen invasion, they are activated and produce a series of antimicrobial peptides (AMPs), including defensins, microplusin, and tick venom proteins. These tick-derived AMPs exhibit antibacterial efficacy against a variety of bacteria, fungi, and certain parasites (Li et al., 2021; Martins et al., 2019; Yu et al., 2006). As a core component of the tick innate immune system, AMP synthesis is significantly induced and upregulated by blood-feeding and microbial infections, acting as a regulated immune response to external microbial stressors (Ceraul et al., 2007; Fogaça et al., 2021). These peptides serve as vital effector molecules with broad-spectrum activity against various microorganisms, helping ticks combat pathogenic infection and sustain normal physiological survival (Wu et al., 2022).

Defensins are typically less than 10 kDa, cationic, amphipathic peptides containing six conserved cysteine residues that form three pairs of intramolecular disulfide bonds, constructing a stable CSαβ fold structure, which is the basis for their stability and activity (Li et al., 2021; Leannec-Rialland et al., 2022; Wu et al., 2022). Previous studies have identified defensins in various tick species and confirmed their inhibitory effects on some bacteria, fungi, and even viruses. For example, Holosin2 and Holosin3 from *Ixodes holocyclus* exhibit significant activity against *Staphylococcus aureus* and *Listeria grayi*, low activity against Gram-negative bacteria such as *Escherichia coli* and *Pseudomonas aeruginosa*, and antifungal activity against *Fusarium graminearum* and *Candida albicans* (Cabezas-Cruz et al., 2019). HEdefensin from *Haemaphysalis longicornis* shows significant antibacterial activity mainly against Gram-positive bacteria (Yada et al., 2018). In addition, persulcatusin from *Ixodes persulcatus* also has potent antibacterial activity against multidrug-resistant *S. aureus* (Miyoshi et al., 2017).

Research on tick immune defense mechanisms helps clarify the molecular basis of tick–pathogen interactions, while also providing clues for the screening of novel bioactive compounds. Multiple defensin genes are upregulated in *H. anatolicum* upon stimulation with common superficial pyogenic bacteria (Li et al., 2025). This study focuses on two newly discovered defensin genes from *H. anatolicum*, conducting analytical and *in vitro* functional verification studies. The aim is to evaluate their antibacterial activity, hemolytic activity, and antioxidant capacity. This study provides reference data for the functional research of *H. anatolicum* defensin genes and offers candidate molecules for the control of tick-borne pathogen transmission and the development of novel, efficient, and low-toxic antimicrobial agents.

## 2 Materials and methods

### 2.1 Experimental animals and tick samples

*H. anatolicum* used in the experiment were artificially reared ticks preserved in the College of Veterinary Medicine, Xinjiang Agricultural University. The experimental conditions for the rearing and storage in the laboratory were maintained as follows: an ambient air temperature of 25 ± 2 °C, a relative humidity of 85% ± 5%, and a photoperiod consisting of 15 h of illumination and 9 h of darkness. All animal procedures were carried out in accordance with the guidelines in the Document 2,023,025 approved by the Animal Ethics Committee of Xinjiang Agricultural University.

### 2.2 Bacterial strains and experimental reagents

Gram-positive bacteria: *S. aureus* (ATCC 6538), *E. faecalis* (ATCC 29212), and *B. subtilis* (CMCC 653501). Gram-negative bacteria: *P. aeruginosa* (ATCC 27853) and *E. coli* (CMCC 44102). All strains were purchased from HaiBo Biotechnology Co., Ltd., Qingdao, China. Molecular biology reagents: The pET-32a^+^ vector and enterokinase were obtained from TransGen Biotech Co., Ltd.; *E. coli* BL21(DE3) competent cells, DNA polymerase, restriction enzymes *Eco*RI and *Xho*I, and T4 DNA ligase were purchased from TaKaRa Biotechnology Co., Ltd.; Ni-NTA resin for His-tagged protein affinity chromatography purification was obtained from Beyotime Biotechnology Co., Ltd., Shanghai, China.

### 2.3 Bioinformatic analysis of Han-Def1 and Han-Def2

Han-Def1 and Han-Def2 were identified based on transcriptome data from our previous study, which are available in the NCBI Gene Expression Omnibus (GEO) database^1^ under accession number GSE325854 (Li et al., 2025). The sequences characterized in this study were submitted to GenBank under accession numbers PX972500 (Han-Def1) and PX972501 (Han-Def2), and homology analysis was conducted using the online BLASTP tool at NCBI. Multiple sequence alignment of Han-Def1 and Han-Def2 with amino acid sequences of defensins from other tick species downloaded from the GenBank database was conducted using Jalview 2.11.5.1 software (Waterhouse et al., 2009). Conserved motif and conserved domain analyses of the defensin proteins were carried out using MEME 5.5.9^2^ and NCBI Batch CD-Search^3^ (Bailey et al., 2015; Wang et al., 2023). ExPASy-ProtParam,^4^ Protscale,^5^ SignalP 6.0,^6^ TMHMM 2.0,^7^ ScanProsite,^8^ PSIPRED,^9^ and AlphaFold3^10^ were used to predict and analyze the basic physicochemical properties, signal peptide cleavage sites, transmembrane regions, disulfide bond positions, secondary structures, and tertiary structures of Han-Def1/Han-Def2 (Abramson et al., 2024; de Castro et al., 2006; Jones, 1999; Krogh et al., 2001; Teufel et al., 2022; Wilkins et al., 1999). Potential disordered regions in the defensin sequences were predicted using IUPred3^11^ with the short disorder prediction mode (Erdős et al., 2021). Residues with a disorder score > 0.5 were considered intrinsically disordered. Amino acid sequences of defensins from different tick species were downloaded from GenBank, aligned using MEGA-X software under default settings, and a phylogenetic tree was constructed by the Maximum-Likelihood method with branch reliability evaluated by 1,000 bootstrap replicates (Kumar et al., 2018). The GenBank accession numbers of all defensin sequences used in this study are provided in Supplementary Table S1.

### 2.4 Cloning and construction of expression vectors for Han-Def1 and Han-Def2 genes

#### 2.4.1 Cloning of the *Han-Def1* and *Han-Def2* genes

Adult female *H. anatolicum* ticks were rinsed with pre-chilled PBS, and total RNA was extracted using the Trizol method (Invitrogen, California, USA). First-strand cDNA was synthesized following the manufacturer’s instructions of the reverse transcription kit. Using the prepared cDNA library as a template, the genes encoding the mature peptides of *Han-Def1* and *Han-Def2* from *H. anatolicum* were amplified. The purified PCR products were cloned into the pMD-19-T vector, and the recombinant plasmid was transformed into *E. coli* DH5α competent cells. The cells were cultured overnight at 37 °C on LB agar plates supplemented with 100 mg/L ampicillin. Single colonies were selected, cultured, and verified by colony PCR. Positive colonies were sent to Sangon Biotech Co., Ltd. for sequencing. Primer sequences and reaction systems are shown in Table S2.

#### 2.4.2 Construction and identification of recombinant plasmids pET32a-Def1 and pET32a-Def2

After analyzing the restriction sites of *Han-Def1*/*Han-Def2* genes and the pET-32a plasmid using DNAMAN v6 software (Lynnon Biosoft, San Ramon, CA, USA), *Eco*RI and *Xho*I were selected as restriction enzyme sites. PCR primers containing *Eco*RI, *Xho*I restriction sites, and an enterokinase cleavage site were designed. Using the positive bacterial liquid as a template, the *Han-Def1/Han-Def2* CDSs were amplified by PCR. The recovered and purified products were digested with *Eco*RI and *Xho*I, and then cloned into the pET-32a vector to construct recombinant plasmids. After verification by PCR, positive recombinant plasmids were sent to Sangon Biotech (Shanghai) Co., Ltd. for sequencing.

### 2.5 Induction expression and purification of recombinant proteins

Recombinant plasmids pET-32a-Def1 and pET-32a-Def2 were transformed into *E. coli* BL21 (DE3) competent cells. The cells were cultured at 37 °C with shaking at 180 rpm for 2–2.5 h until the OD₆₀₀ value reached 0.4–0.6. IPTG was added to a final concentration of 1 mmol/L, and induction was continued at 37 °C with shaking at 180 rpm for 10 h. The induced culture was harvested and centrifuged at 10,000 rpm for 10 min at 4 °C, and the supernatant was discarded. The cell pellet was resuspended in non-denaturing lysis buffer and disrupted by sonication in an ice bath under the following conditions: 4 °C, 25% power, 3 s sonication, 5 s interval, for a total of 40 min. After sonication, the supernatant was collected by centrifugation at 12,000 rpm for 20 min at 4 °C. The recombinant proteins rDef1 and rDef2 were purified using a Ni-column resin for His-tagged protein purification. The expression and purification of the two proteins were analyzed by SDS-PAGE. Protein concentration was determined using a BCA Protein Assay Kit (Solarbio Science & Technology Co., Ltd., Beijing, China).

### 2.6 Cleavage of the fusion tag and recovery of the target protein

The purified protein samples were dialyzed against 25 mM Tris–HCl buffer (pH 8.0). Recombinant enterokinase (100 μg/2 U, Solarbio Science & Technology Co., Ltd., Beijing, China) was added according to the fusion protein concentration, and digestion was performed overnight at 25 °C to separate the fusion tag from the target protein. The digested products were subjected to ultrafiltration and centrifugation using ultrafiltration tubes to obtain high-purity defensins.

### 2.7 Antimicrobial activity assay

The minimum inhibitory concentration (MIC) and minimum bactericidal concentration (MBC) of tag-free rDef1 and rDef2 against *S. aureus*, *E. faecalis*, *B. subtilis*, *P. aeruginosa* and *E. coli* were determined using the twofold microdilution method. Ampicillin and Gentamicin were included as positive antibiotic controls for Gram-positive bacteria (*S. aureus*, *E. faecalis*, and *B. subtilis*) and Gram-negative bacteria (*P. aeruginosa* and *E. coli*), respectively. Briefly, bacterial strains were cultured to mid-log phase and diluted to approximately 1 × 10^6^ CFU/mL. In a sterile 96-well microplate, 100 μL of Mueller-Hinton (MH) broth was dispensed into wells 2 to 6. Then, 200 μL of the defensin stock solution was placed into the first well. A twofold serial dilution was performed by transferring 100 μL from well 1 to well 2, mixing thoroughly, and then sequentially transferring 100 μL from each well to the next, up to well 6, from which 100 μL was discarded after mixing. Subsequently, 100 μL of the bacterial suspension (1 × 10^6^ CFU/mL) was added to each well, resulting in a final inoculum of approximately 5 × 10^5^ CFU/mL. Under these conditions, the final tested concentration ranges were 750–23 μg/mL (182–5.6 μM) for rDef1 and 850–26 μg/mL (199–6.1 μM) for rDef2. Well 7 (medium + bacteria) served as the growth control, and well 8 (medium only) as the blank control. Additionally, a buffer control containing bacteria and an equivalent volume of PBS was included to verify that the solvent did not affect bacterial growth. After incubation at 37 °C for 15 h, 10 μL of 5 g/L TTC was added to each well, followed by further incubation for 3 h. The MIC was defined as the lowest concentration with no visible red color. All assays were performed in triplicate, and consistent results were obtained across three independent experiments (Wiegand et al., 2008). For MBC determination, aliquots from clear wells were plated onto MH agar. The MBC was defined as the concentration yielding fewer than 5 colonies (Sarker et al., 2007).

### 2.8 Hemolytic activity assay

To verify the hemolytic activity of the recombinant proteins against sheep red blood cells, laying a foundation for future *in vivo* studies in sheep and providing a reference for safety evaluation, hemolytic assays of the recombinant proteins were performed in this study (Chatupheeraphat et al., 2023).

Fresh sheep blood was collected into EDTA-containing anticoagulant tubes and kept on ice. The blood was centrifuged at 1,000 × g for 10 min at 4 °C, and the plasma and buffy coat were discarded. The packed erythrocytes were washed three times with sterile 0.9% normal saline and then resuspended in the same buffer to obtain a 4% (v/v) erythrocyte suspension. Recombinant defensins rDef1 and rDef2 were diluted to the same concentration and subjected to two-fold serial dilutions in sterile 0.9% normal saline. Each dilution was then mixed 1:1 with an equal volume of 4% (v/v) erythrocyte suspension. The final concentration range were 750–23 μg/mL (182–5.6 μM for rDef1 and 175–5.4 μM for rDef2), and the final concentration of erythrocytes was 2% (v/v).

After incubation at 37 °C for 1 h, the mixtures were centrifuged at 1,000 rpm for 10 min, and the supernatants were collected for absorbance measurement at 540 nm. Each treatment concentration was assayed using three independent replicates. The hemolysis rate was calculated using the following formula, and the average value was recorded:


$$\text{Hemolysis rate}\;(\%)=\frac{OD_{\text{sample}}-OD_{\text{negative}}}{OD_{\text{positive}}-OD_{\text{negative}}}\times 100$$

Where OD_sample_ = absorbance of the protein sample group, OD_negative_ = absorbance of the normal saline group, OD_positive_ = absorbance of the 1% Triton X-100 group. The final hemolysis rates are reported as mean ± standard deviation (SD) from three independent experiments.

### 2.9 Antioxidant activity analysis

The antioxidant activities of rDef1 and rDef2 were evaluated *in vitro* (Wang et al., 2017). Purified rDef1 and rDef2 were first diluted to the same concentration, then serially two-fold diluted in PBS to obtain a concentration range of 1,500–23 μg/mL (364–5.6 μM for rDef1 and 350–5.4 μM for rDef2). The scavenging capacity for ABTS free radical, as well as total antioxidant capacity (TAC) of rDef1 and rDef2, was determined using commercial kits (Grace Biotechnology Co. Ltd., Suzhou, China) according to the manufacturer’s instructions, and Trolox solution was used as the positive control. A standard curve was constructed using Trolox standards (0–100 μg/mL), and the linear regression equation obtained was:

y = 0.5042x − 1.4213(R^2^ = 0.9971).

The ABTS free radical scavenging rate and total antioxidant capacity (TAC) were calculated using Equations 1, 2, respectively.


$$\text{ABTS free radical scavenging rate}\;(\%)=[1-\frac{A_{s}-A_{c}}{A_{b}}]\times 100$$ (1)

$$\mathrm{TAC}\;(\mathrm{\mu g}\;\text{Trolox}/\mathrm{mL})=\frac{\text{scavenging rate}+1.4213}{0.5042}\times D$$ (2)

In Equations 1, 2, A_s_ is the absorbance value of the rDef1 or rDef2 sample group, A_c_ is the absorbance value of control groups, A_b_ is the absorbance value of blank groups, and D is the dilution ratio, with undiluted being 1-fold. All determinations were performed in three independent replicates.

### 2.10 Statistical analysis

All experiments were performed in at least triplicate, and data are expressed as mean ± standard deviation (SD). Statistical analyses were performed using GraphPad Prism 8.0. Differences between rDef1 and rDef2 groups across different concentrations were analyzed using two-way analysis of variance (ANOVA) followed by Sidak’s multiple comparison test. A *p*-value of < 0.05 was considered statistically significant. Levels of significance are indicated as follows: *p < 0.05, **p < 0.01, and ***p < 0.001.

## 3 Results

### 3.1 Bioinformatic characterization of Han-Def1 and Han-Def2

#### 3.1.1 Prediction of protein structure and physicochemical properties of Han-Def1 and Han-Def2

**Figure 1:**
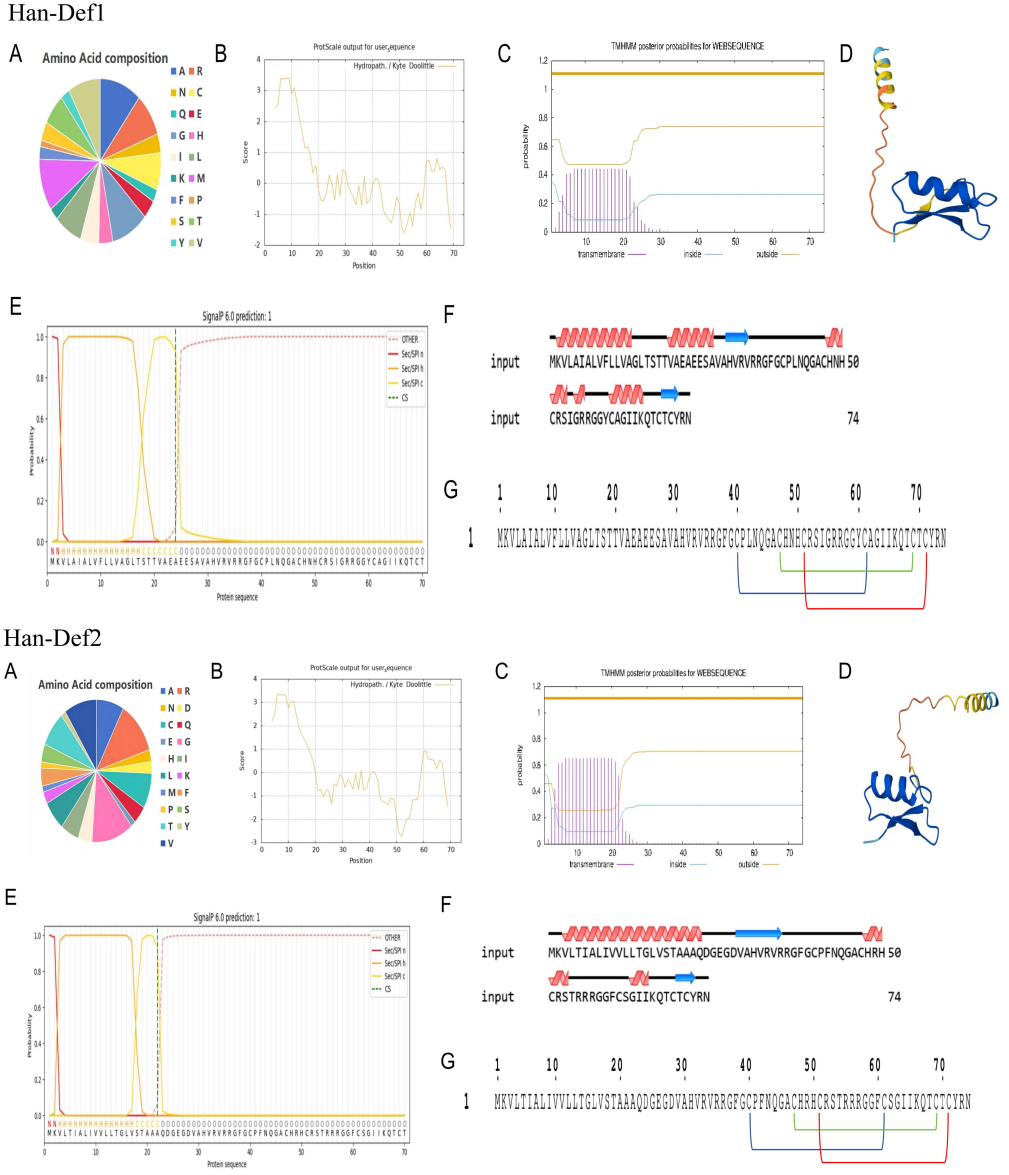
Prediction results of the structure and physicochemical properties of Han-Def1 and Han-Def2 protein. **(A)** Prediction and analysis of amino acid composition. **(B)** Prediction and analysis of hydrophilicity and hydrophobicity. **(C)** Transmembrane structure prediction. **(D)** Three-dimensional structural modeling. **(E)** Signal peptide prediction. **(F)** Secondary structure prediction. **(G)** Prediction of disulfide bond position.

The physicochemical properties of the amino acid sequences of Han-Def1 and Han-Def2 were predicted using the online tool ExPASy-ProtParam. The analysis results showed that Han-Def1 consisted of 74 amino acids with a relative molecular weight of approximately 7.92 kDa and a theoretical isoelectric point (pI) of 9.38, indicating that it is a strongly cationic peptide. Han-Def2 was also composed of 74 amino acids, with a relative molecular weight of about 8.03 kDa and a theoretical pI of 9.99, exhibiting cationic properties as well. Amino acid composition analysis revealed that Han-Def1 and Han-Def2 are composed of 18 and 19 different types of amino acids, respectively. Hydrophobic amino acids accounted for 51.5 and 48.9% of Han-Def1 and Han-Def2, respectively. Han-Def1 contained 9 positively charged residues (Arg + Lys), while Han-Def2 contained 11. Based on the hydrophilicity coefficients (Han-Def1: 0.253; Han-Def2: 0.047), both were classified as weakly hydrophobic proteins. Analysis using the Protscale tool showed that Han-Def1 exhibited the highest hydrophobicity (maximum value: 3.411) at the 6th and 9th amino acid positions, and the lowest hydrophobicity (minimum value: −1.600) at the 52nd amino acid position. In contrast, Han-Def2 displayed the highest hydrophobicity (maximum value: 3.367) at the 6th amino acid position and the lowest hydrophobicity (minimum value: −2.744) at the 52nd amino acid position. The N-terminal regions of both proteins exhibited high hydrophobicity, a conserved feature of secretory signal peptides that facilitates essential membrane interactions during protein translocation and secretion (Figure 1).

The SignalP 6.0 online server was used to predict signal peptides. The results indicated that obvious signal peptide cleavage sites existed at the N-terminus of the precursor proteins of both Han-Def1 and Han-Def2, located near the 24th (Ala24↓Glu25) and 22nd (Ala22↓Gln23) amino acid positions, respectively, with significant peak values. This suggested that both are secretory proteins, and cleavage of the signal peptide could effectively improve the expression level of recombinant proteins in the prokaryotic system (Dai et al., 2005). Therefore, the signal peptides of Han-Def1 and Han-Def2 were truncated in this study to enhance their expression level and solubility. Transmembrane domain prediction was performed using TMHMM 2.0, and the results excluded the presence of transmembrane helices in both Han-Def1 and Han-Def2, confirming that they are soluble secretory proteins. The secondary structure was predicted by PSIPRED. In the amino acid sequence of Han-Def1, *α*-helix accounted for approximately 37.84%, *β*-sheet accounted for about 8.11%, and the rest were random coils. The secondary structure composition of Han-Def2 was similar to that of Han-Def1, with α-helix accounting for approximately 40.54% and β-sheet accounting for about 8.11%. This combination of “α-helix + antiparallel β-sheet” is a typical feature of forming the cysteine-stabilized α/β (CSαβ) motif. Further sequence analysis using the ScanProsite server confirmed that both Han-Def1 and Han-Def2 sequences contained six highly conserved cysteine (Cys) residues. These Cys residues are expected to form three pairs of intramolecular disulfide bonds in a specific pairing mode (Cys1-Cys4, Cys2-Cys5, Cys3-Cys6), which are crucial for maintaining the stable three-dimensional structure and antibacterial activity of defensins (Figure 1).

Finally, the high-precision three-dimensional structures of the mature peptides of Han-Def1 and Han-Def2 were predicted using the AlphaFold3 platform. The predicted models had high confidence, and their predicted Local Distance Difference Test (pLDDT) values showed that the structure prediction of the core region was highly reliable (pLDDT > 90). As shown in the models (Figure 1), both folded into a compact globular structure, presenting a typical defensin cysteine-stabilized α/β (CSαβ) folding pattern: an amphiphilic α-helix and an antiparallel double-stranded β-sheet were closely cross-linked by three pairs of intramolecular disulfide bonds (Cys1-Cys4, Cys2-Cys5, Cys3-Cys6) to form a stable structural core (Figure 1). Consistently, IUPred3 analysis of the Han-Def1 and Han-Def2 sequences showed that nearly all residues had disorder scores below 0.5, with only the C-terminal residue of Han-Def2 scoring above 0.5, indicating the absence of extensive intrinsically disordered regions (Supplementary Figure S1). Such terminal flexibility is common in small folded proteins and does not affect the overall compact CSαβ fold. This finding further supports the predicted structure and suggests that both defensins are fully structured. Based on all the above analyses, Han-Def1 and Han-Def2 were identified as two typical cationic defensins with stable CSαβ folding structures in *H. anatolicum*, and their shared core folding pattern ensures stability and basic functions.

#### 3.1.2 Multiple sequence alignment

**Figure 2:**
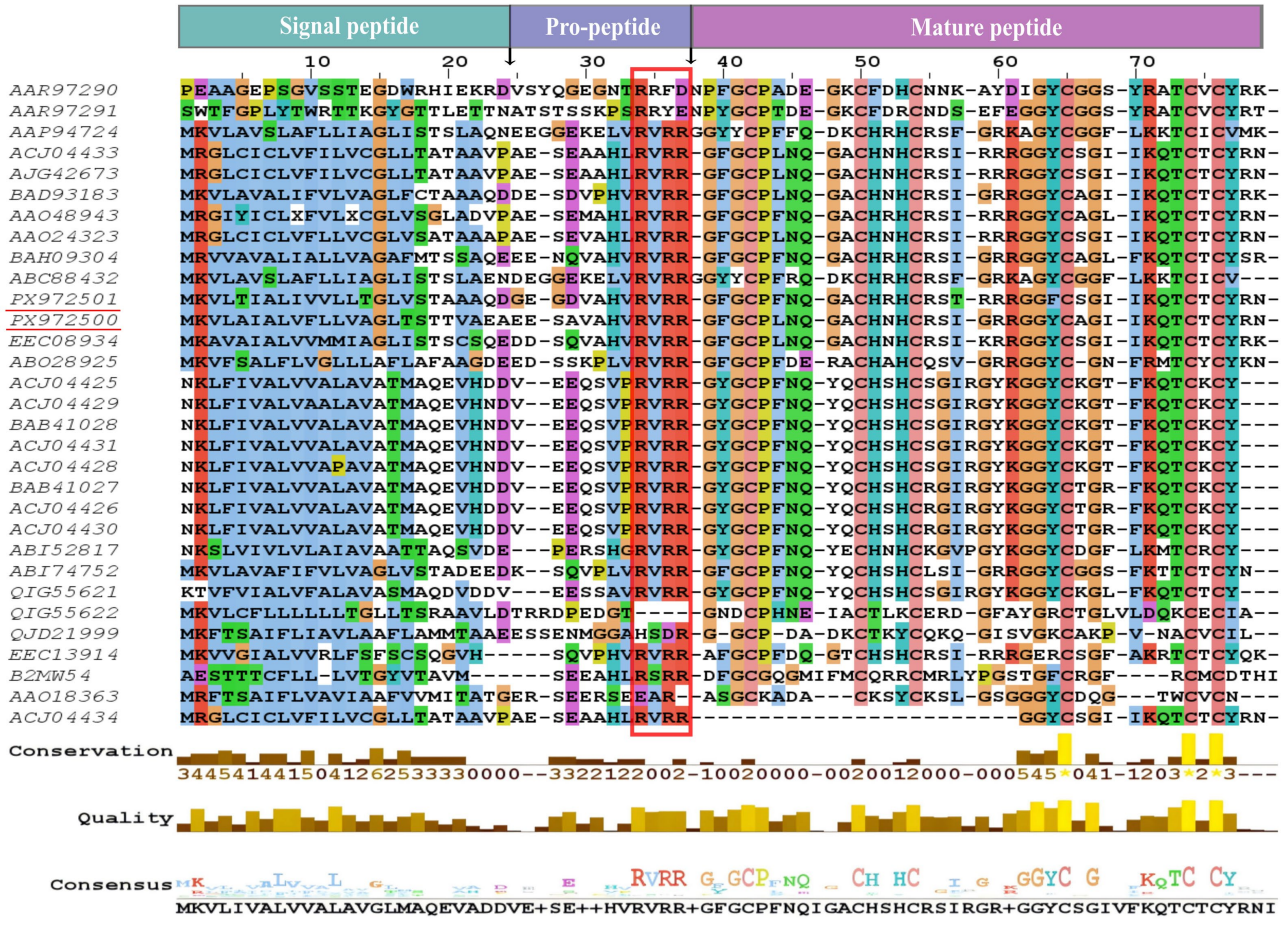
Multiple sequence alignment of *H. anatolicum* defensins with other tick defensins. Amino acid sequences of Han-Def1, Han-Def2, and defensins from other tick species were aligned using Jalview 2.11.5.1. The predicted signal peptide, propeptide, and mature peptide regions are represented in the boxes above the MSA, and the cleavage sites between these regions are indicated by arrows. The mature peptide cleavage site motif RXXR is outlined by a red rectangle. Conserved cysteines and glycines are highlighted in light red and golden, respectively.

The amino acid sequences of Han-Def1 and Han-Def2 were subjected to multiple sequence alignment with defensins from other known tick species (Figure 2). The results showed that both sequences shared high conservation with defensins of the family Ixodidae. The most prominent feature was that the positions of the six cysteine (Cys) residues were fully conserved in Han-Def1, Han-Def2, and all aligned defensins, forming the classical spacing pattern C-X6-C-X3-C-X9-C-X7-C-X-C, which is the essential backbone for the formation of three pairs of intramolecular disulfide bonds (Cys1-Cys4, Cys2-Cys5, Cys3-Cys6) to stabilize the three-dimensional structure (Cabezas-Cruz et al., 2019).

In addition to cysteines, the alignment revealed other key conserved residues. Several glycine (Gly) sites were highly conserved, whose small side chains may provide the necessary conformational flexibility for the compact CSαβ fold of defensins. Furthermore, several positively charged amino acids as well as hydrophobic/aromatic amino acids were also present at conserved positions. The former may mediate the initial binding to negatively charged microbial membranes, while the latter may contribute to maintaining the hydrophobic core of the protein or interactions with membrane lipids.

It was also observed from the alignment that Han-Def1 and Han-Def2 exhibited individual amino acid differences in the C-terminal region, which usually contains the functional *γ*-core motif. Such sequence variations may lead to subtle differences in charge distribution, hydrophobicity, or loop flexibility between the two peptides, potentially resulting in divergence in their antimicrobial spectra or efficacy.

Taken together, multiple sequence alignment confirmed that Han-Def1 and Han-Def2 possess all the core characteristics of the tick defensin family, while variations in their non-conserved regions provide a structural basis for functional diversity.

#### 3.1.3 Phylogenetic, conserved domain and conserved motif analyses of Han-Def1 and Han-Def2

**Figure 3:**
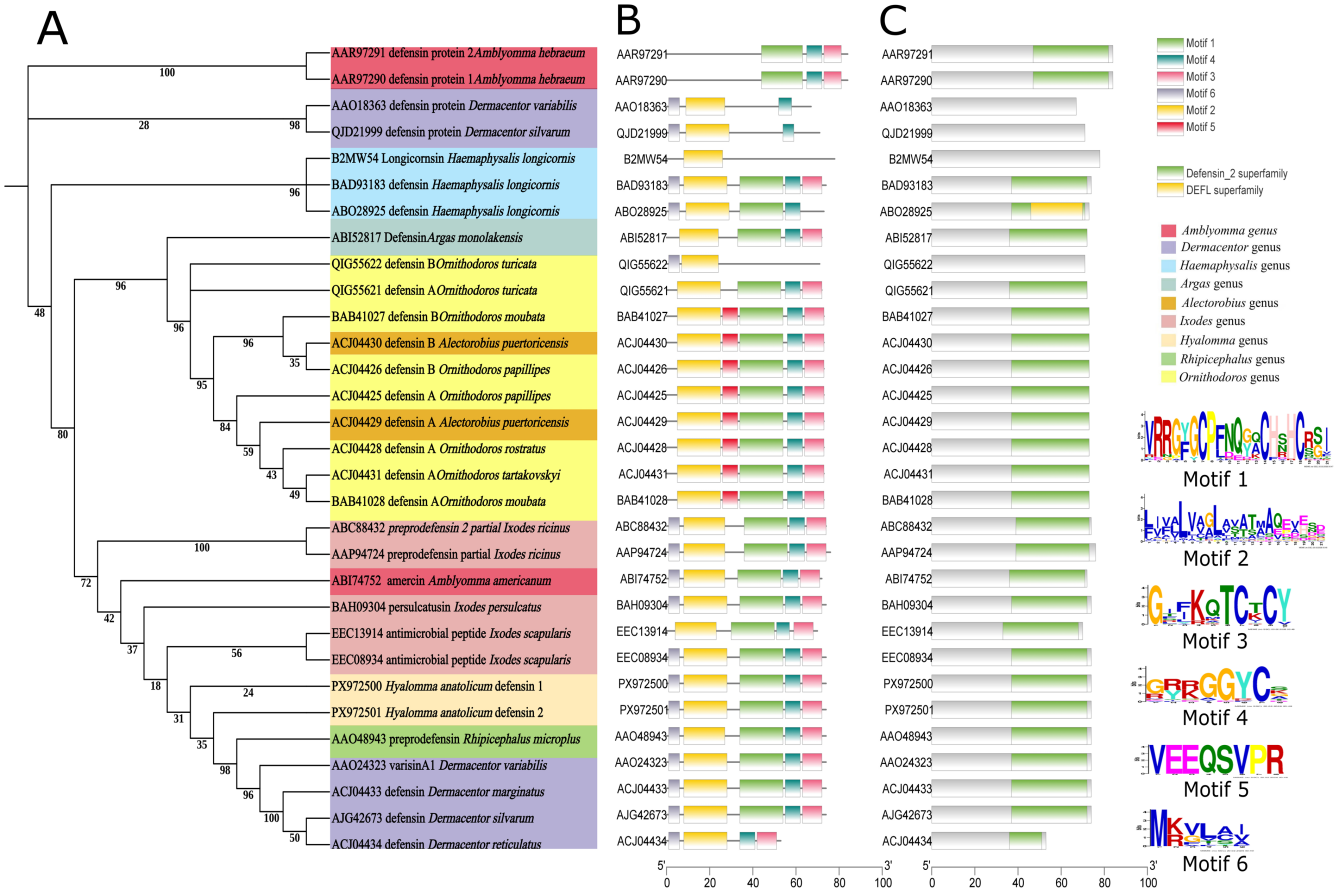
Phylogenetic relationship and conserved characteristics analysis of Han-Def1/Han-Def2 defensins from *H. anatolicum*. **(A)** Phylogenetic tree constructed using the Maximum-Likelihood method based on the defensin sequences. Bootstrap values from 1,000 replicates are shown at the nodes. **(B)** Distribution pattern of six conserved motifs (Motif 1–6) identified by MEME analysis across the defensin sequences. Motif 6 is specific to hard tick defensins. **(C)** Protein domain composition analyzed by NCBI CD-Search, showing the predominant Defensin_2 superfamily domain.

To investigate the evolutionary relationships and sequence characteristics of Han-Def1 and Han-Def2, phylogenetic analysis, conserved domain analysis and conserved motif analysis were performed. Phylogenetic tree construction based on amino acid sequences (Figure 3A) showed that Han-Def1 and Han-Def2 branched within the lineage of ixodid defensins, demonstrating a close evolutionary relationship with other known tick defensins, which was consistent with species taxonomy. The phylogenetic tree was clearly divided into five clades. Defensins of the family Argasidae mainly formed one distinct clade, while defensins of the family Ixodidae were separated into one large clade and three small clades. Some defensins from *Dermacentor*, *Amblyomma* and *Haemaphysalis* were distributed in different branches, suggesting high diversity among these defensins. Han-Def1 and Han-Def2 were clearly assigned to the Ixodid defensin family. Furthermore, they were grouped into the same evolutionary cluster as defensins from *Dermacentor*, *Rhipicephalus* and *Ixodes*, indicating close evolutionary relatedness among these defensins.

Conserved domain analysis was conducted using the NCBI Conserved Domain Database (CDD) for homologous sequences including Han-Def1 and Han-Def2. Most sequences (27 out of 31) contained a typical Defensin 2 superfamily domain (Figure 3C). This domain is an important taxonomic unit in antimicrobial peptides, and in tick defensins it usually corresponds to the cysteine-stabilized *α*/*β* (CSαβ) folding core stabilized by three pairs of disulfide bonds (Wu et al., 2022). The universal existence of this domain confirmed that these peptides belong to the defensin superfamily at the functional unit level. In addition, a DEFL superfamily domain was identified in only one sequence, which may represent a specific variant subgroup in the defensin family. Notably, no known domain was detected in four sequences, which may result from high sequence divergence or location at the boundary of known defensin domains. Overall, the Defensin 2 superfamily domain represents the main conserved functional domain of Han-Def1, Han-Def2 and related tick defensins, supporting their typical CSαβ folding structure and antimicrobial activity.

Conserved motif analysis further revealed the fine characteristics of the sequences (Figure 3B). Conserved motif analysis revealed six conserved motifs (Motif 1–6). Motif 1, 2, 3 and 4 were widely distributed in tick defensins, Motif 5 was present only in defensins of Argasidae, and Motif 6 was unique to defensins of Ixodidae. In the amino acid sequences of Han-Def1 and Han-Def2, the positions of six cysteine (Cys) residues were completely conserved and mainly distributed in Motif 1, Motif 3 and Motif 4, forming the typical spacing pattern of defensins (C-X6-C-X3-C-X9-C-X7-C-X-C).

### 3.2 Expression, purification and cleavage of rHan-Def1 and rHan-Def2

**Figure 4:**
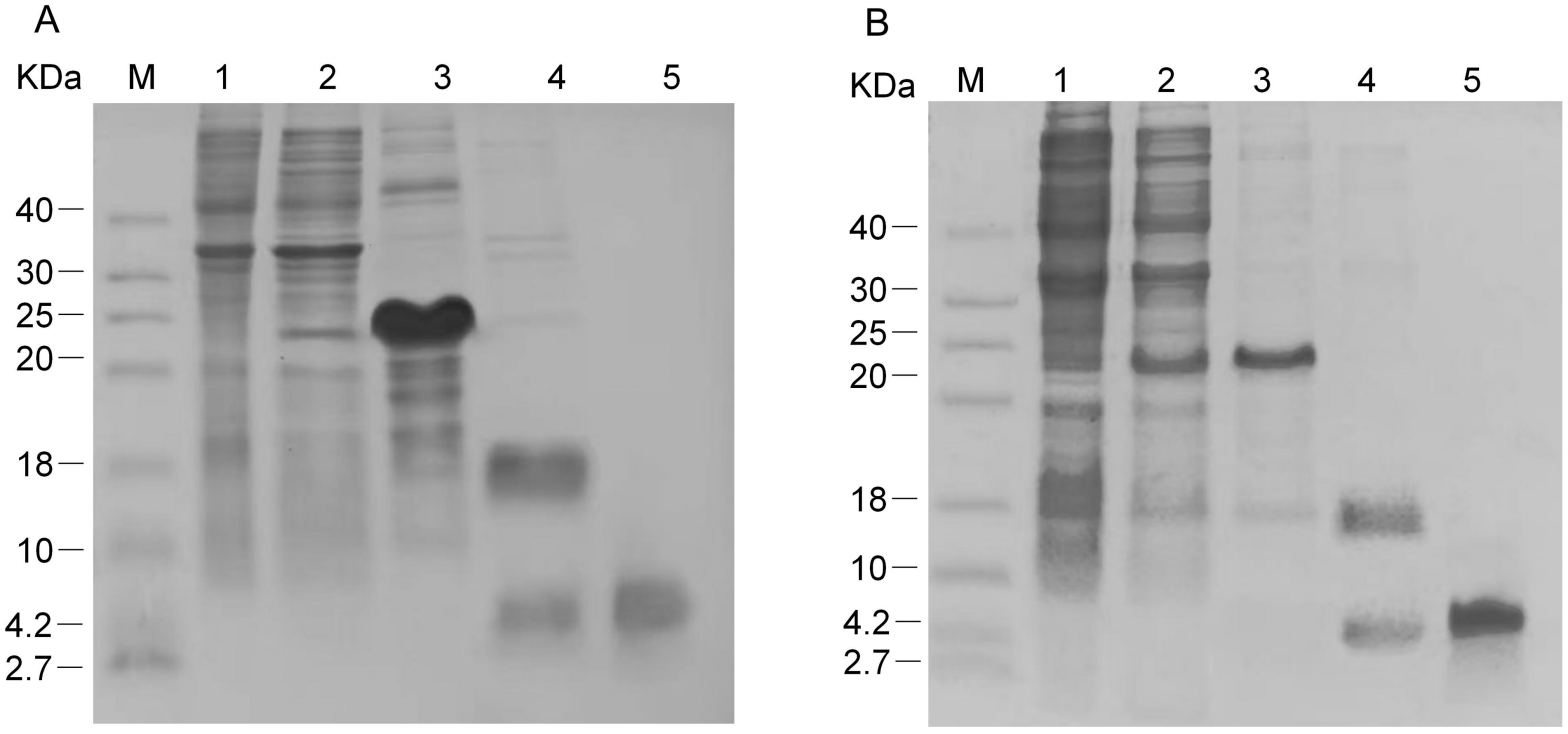
SDS-PAGE analysis of recombinant rDef1 **(A)** and rDef2 **(B)** expression and purification. Lane M: Protein molecular weight marker. Lane 1: Total cell lysate before IPTG induction. Lane 2: Total cell lysate after IPTG induction, showing expression of the Trx-His-tagged fusion protein. Lane 3: Purified fusion protein after nickel-affinity chromatography. Lane 4: Digestion products of the fusion protein with enterokinase. Lane 5: Tag-free mature defensin obtained after ultrafiltration concentration.

After bioinformatics-based identification, the mature peptide coding sequences of Han-Def1 and Han-Def2 were successfully cloned and directionally inserted into the pET-32a^+^ vector. Sequencing and restriction enzyme digestion confirmed the correct construction of the recombinant expression plasmids, pET-32a-EK-rDef1 and pET-32a-EK-rDef2 (Supplementary Figures S2–S4). The recombinant plasmids were transformed into *E. coli* BL21 (DE3) strain, and induced expression was performed with IPTG at a final concentration of 1.0 mmol/L, followed by purification. SDS-PAGE analysis showed that after IPTG induction, obvious target protein bands appeared at approximately 22 kDa in the lysis supernatant of broken rDef1 and rDef2, respectively (Figure 4). The size was consistent with the expected value, and the recombinant proteins were mainly expressed in a soluble form.

Nickel column resin for His-tagged protein purification was used to purify the fusion proteins, and high-purity rHan-Def1 and rHan-Def2 were obtained (Figure 4). To obtain defensins with a structure close to the natural state, enterokinase was used to cleave the purified fusion proteins and remove the Trx-His tag. After the cleavage products were separated by ultrafiltration and centrifugation, SDS-PAGE detection showed a single main band around 4 kDa (Figure 4, lane 5). Notably, this molecular weight was significantly lower than that of the fusion proteins (approximately 22 kDa, Figure 4, lane 3), and was consistent with the theoretical molecular weight of the mature peptides of Han-Def1 and Han-Def2, confirming the successful removal of the Trx-His tag. Finally, 5 mL of purified tag-free mature peptide solution was obtained, with final concentrations determined to be approximately 1,500 μg/mL (364 μM) for rDef1 and 1700 μg/mL (397 μM) for rDef2, respectively, using a BCA Protein Assay Kit (Solarbio Science & Technology Co., Ltd., Beijing, China). This corresponds to a total yield of approximately 18.75 mg/L for rDef1 and 21.25 mg/L for rDef2, providing sufficient material for the subsequent biological activity analysis.

### 3.3 Antimicrobial activity of rDef1 and rDef2

The MIC and MBC values of rDef1 and rDef2 against five representative pathogenic isolates are summarized in Table 1. To validate the susceptibility profile of the tested strains, Ampicillin and Gentamicin were used as positive controls. Ampicillin exhibited potent inhibitory activity against Gram-positive strains (*S. aureus* and *E. faecalis*: 3.12 μg/mL, 8.9 μM; *B. subtilis*: 6.25 μg/mL, 17.9 μM), while Gentamicin was highly effective against Gram-negative isolates (*P. aeruginosa*: 0.78 μg/mL, 1.6 μM; *E. coli*: 1.56 μg/mL, 3.3 μM). These results confirm that the bacterial isolates used in this study were antibiotic-sensitive standard strains.

**Table 1:** MIC and MBC values of rDef1 and rDef2 against five bacterial strains.

Bacteria	MIC (μg/mL [μM])		MBC (μg/mL [μM])	
	Def1	Def2	Def1	Def2
Gram-positive bacteria				
*Staphylococcus aureus* (ATCC 6538)	46 [11.2]	106 [24.8]	46 [11.2]	106 [24.8]
*Enterococcus faecalis* (ATCC 29212)	93 [22.6]	425 [99.3]	186 [45.1]	850 [199]
*Bacillus subtilis* (CMCC 653501)	750 [182]	850 [199]	750 [182]	850 [199]
Gram-negative bacteria				
*Pseudomonas aeruginosa* (ATCC 27853)	No effect	No effect	No effect	No effect
*Escherichia coli* (CMCC 44102)	No effect	No effect	No effect	No effect

Both rDef1 and rDef2 exhibited selective antimicrobial activity favoring Gram-positive bacteria. rDef1 demonstrated superior potency, with MIC and MBC values against *S. aureus* of 46 μg/mL (11.2 μM). It also showed significant inhibitory effects on *E. faecalis* (93 μg/mL, 22.6 μM) and *B. subtilis* (750 μg/mL, 182 μM). In contrast, rDef2 showed a similar but weaker inhibitory trend, with MIC values of 106 μg/mL (24.8 μM), 425 μg/mL (99.3 μM), and 850 μg/mL (199 μM) against *S. aureus*, *E. faecalis,* and *B. subtilis*, respectively. Notably, neither rDef1 nor rDef2 exhibited detectable activity against the Gram-negative pathogens *P. aeruginosa* and *E. coli* within the tested concentration range, suggesting a distinct functional preference for Gram-positive cell wall structures.

### 3.4 Hemolytic activity of rDef1 and rDef2

**Figure 5:**
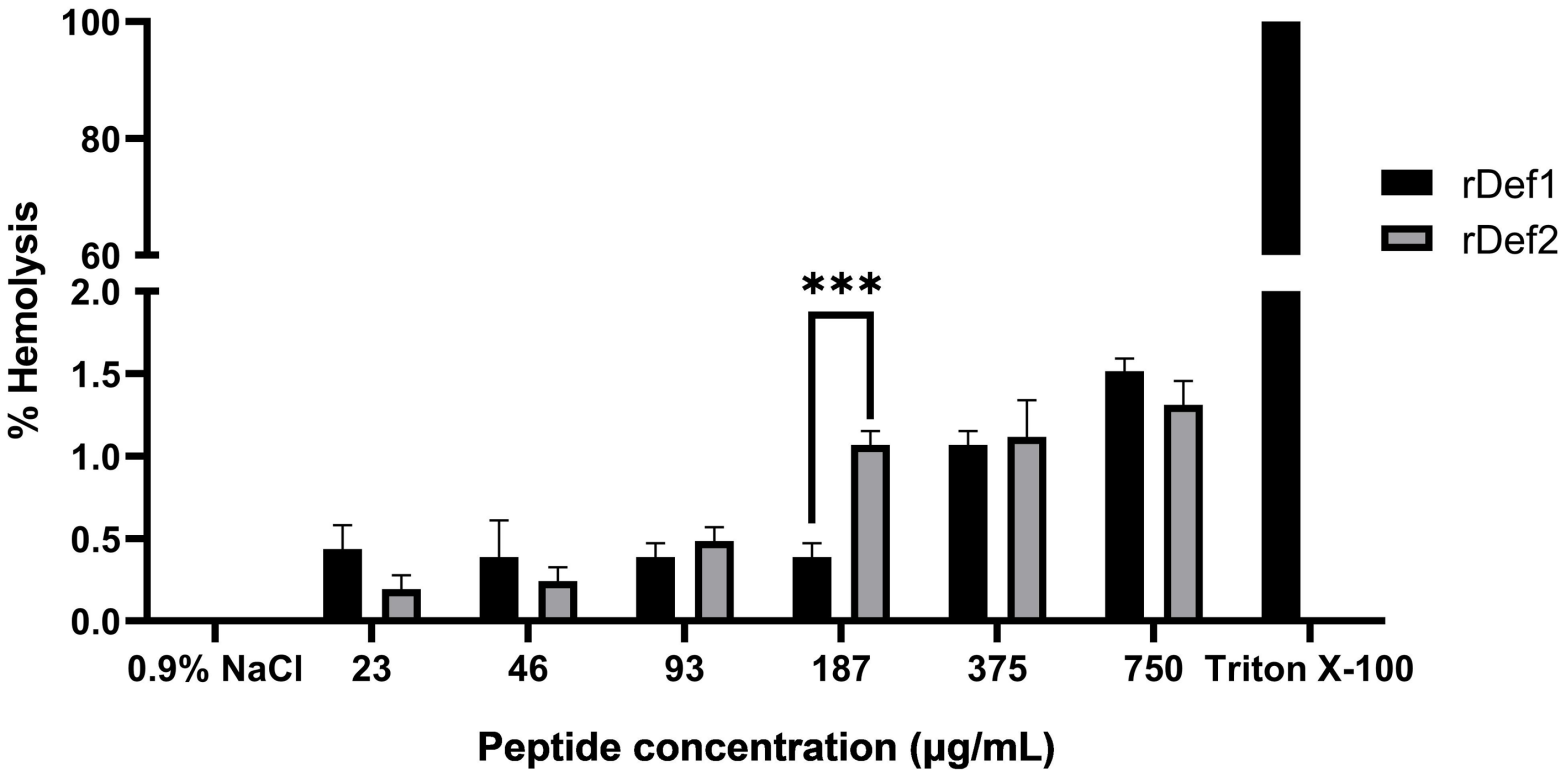
Hemolytic activity of rDef1 and rDef2 against sheep erythrocytes. Sheep erythrocytes were incubated with serially diluted rDef1 or rDef2 at final concentrations ranging from 750 to 23 μg/mL (182–5.6 μM for rDef1 and 175–5.4 μM for rDef2). Hemolysis was quantified by measuring the absorbance of the supernatant at 540 nm. Normal saline and 1% Triton X-100 were used as the negative and positive controls, respectively. Data are presented as mean ± SD from three independent experiments. Statistical significance was analyzed between rDef1 and rDef2 at the same concentration. *p < 0.05, ***p < 0.001.

To evaluate the safety of rDef1 and rDef2 to mammalian cells, their hemolytic activity against sheep red blood cells was determined. At the maximum tested final concentrations (750 μg/mL, corresponding to 182 μM for rDef1 and 175 μM for rDef2), the hemolysis rates were negligible, measured at 1.51 and 1.31%, respectively (Figure 5). Since hemolysis did not reach 50% even at the highest concentrations tested, the HC50 values were determined to be > 750 ug/mL for rDef1 and rDef2, indicating negligible hemolytic toxicity. In contrast, the positive control (1% Triton X-100) caused 100% hemolysis. These results indicated that rDef1 and rDef2 had high selectivity for eukaryotic cell membranes within the effective antimicrobial concentration range, showing good biocompatibility.

### 3.5 Antioxidant activity of rDef1 and rDef2

**Figure 6:**
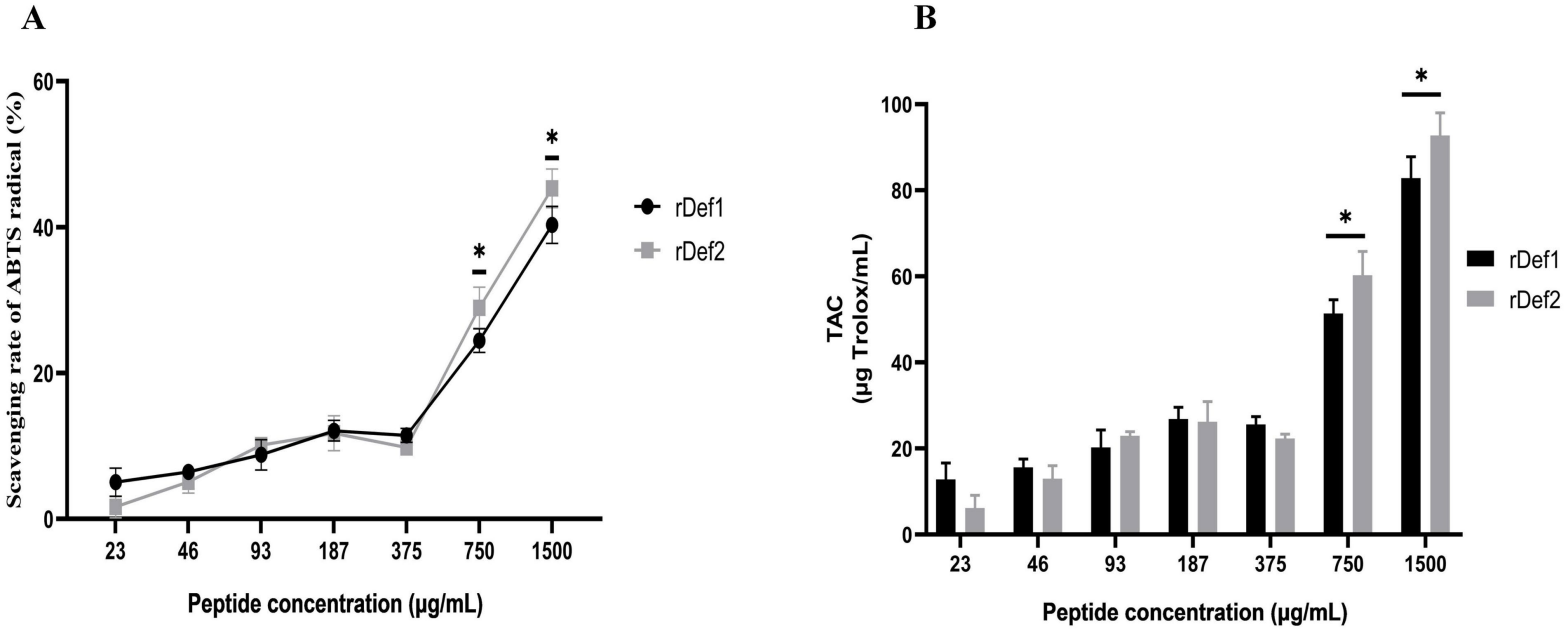
ABTS radical scavenging ability of rDef1 and rDef2. **(A)** ABTS radical scavenging rate of rDef1 and rDef2 at final concentrations ranging from 1,500 to 23 μg/mL (364–5.6 μM for rDef1 and 350–5.4 μM for rDef2). **(B)** Total antioxidant capacity (TAC) of rDef1 and rDef2 expressed as Trolox equivalents. Trolox was used as the positive control. Data are presented as mean ± SD from three independent experiments. Statistical significance was analyzed between rDef1 and rDef2 at the same concentration. *p < 0.05.

The *in vitro* antioxidant capacity of the purified rDef1 and rDef2 was evaluated using the ABTS free radical scavenging method (Figure 6). The results showed that the ABTS scavenging rate of rDef1 was 40.3% at a concentration of 1,500 μg/mL (364 μM), while that of rDef2 was 45.3% at 1500 μg/mL (350 μM), indicating that both rDef1 and rDef2 possessed free radical scavenging activity (Figure 6A). The ABTS scavenging capacity, expressed as Trolox equivalents, was specifically higher for rDef2 (92.75 μg Trolox/mL) than for rDef1 (82.83 μg Trolox/mL) (Figure 6B). Although their antioxidant capacity was weaker than that of classic small-molecule antioxidants, this result indicated that in addition to direct antimicrobial function, Han-Def1 and Han-Def2 may also play an auxiliary role in alleviating oxidative stress in the immune defense of ticks or their interaction with hosts through their antioxidant activity.

## 4 Discussion

Defensins are a class of antimicrobial peptides widely present in insects, ticks, and other invertebrates, with broad-spectrum antibacterial, antiviral, and antiparasitic activities (Boman, 1995; Mansfield et al., 2017). As cationic antimicrobial peptides, defensins exert their biological functions through multiple mechanisms, such as disrupting pathogen cell membranes and inhibiting pathogen proliferation, and play important roles in the immune control of microbial infections as natural effectors and immunomodulators (Wu et al., 2022). In recent years, with the increasing prevalence of antibiotic resistance, the development of novel antimicrobial agents has become a global focus. Natural antimicrobial peptides, especially tick-derived defensins, are considered potential antibiotic substitutes and biological control tools due to their characteristics of high efficiency, low toxicity, and low tendency to induce drug resistance (Li et al., 2021).

Previous studies have explored to apply tick defensins to the field of biological control. For example, Ds-defensin, a defensin isolated from the hard tick *Dermacentor sil*var*um*, exhibits potent inhibitory activity against a variety of Gram-positive bacteria and fungi, and shows no hemolytic or cytotoxic activity at the tested concentrations, making it a promising safe antimicrobial agent for mammalian systems (Wang et al., 2015). Defensins holosins 2 and 3 identified from the Australian paralysis tick *I. holocyclus* also have inhibitory effects on both Gram-positive and Gram-negative bacteria, further demonstrating the potential of tick defensins in combating bacterial infections (Cabezas-Cruz et al., 2019). Derived peptides of tick defensins have also shown certain application prospects in agricultural biological control. TickCore3 (TC3), a linear peptide designed based on the *γ*-core region of the tick defensin DefMT3, can significantly inhibit the growth of *Fusarium graminearum*, an important crop pathogenic fungus, and efficiently block the production of type B trichothecene mycotoxins, which provides a new idea for the development of environment-friendly prevention and control strategies for fungal diseases (Leannec-Rialland et al., 2021).

In the present study, two defensins (Han-Def1 and Han-Def2) from *H. anatolicum* were characterized to possess the molecular characteristics and biological potential of novel antimicrobial peptides through systematic bioinformatics analysis, recombinant expression and functional verification. Multiple sequence alignment and phylogenetic analysis indicated that the two defensin molecules have the core characteristics of tick defensins: six completely conserved cysteine residues, whose spacing pattern (C-X6-C-X3-C-X9-C-X7-C-X-C) is highly consistent with the reported defensins of Ixodidae (Tonk et al., 2014a), and intramolecular disulfide bonds form the backbone of a stable three-dimensional structure (Talactac et al., 2017). The globular structure and typical cysteine-stabilized *α*/*β* (CSαβ) folding pattern of Han-Def1 and Han-Def2 are widely present in insect and tick defensins, which may theoretically resist protease degradation and may maintain biological activity for a relatively long time (Wu et al., 2022).

Prediction of physicochemical properties showed that Han-Def1 and Han-Def2 are typical cationic defensins, which is consistent with the initial step of the mechanism by which most antimicrobial peptides target negatively charged microbial membranes through electrostatic interaction (Sagaram et al., 2011; Tonk et al., 2014b). Previous studies have shown that the net charge of defensins has a significant impact on their antimicrobial efficacy, among which the increase in the number of positive charge residues is positively correlated with the improvement of antimicrobial efficacy, while the effect of negative charge residues is the opposite (Raj et al., 2000; Taylor et al., 2010). Meanwhile, the proportions of hydrophobic amino acid residues in Han-Def1 and Han-Def2 are as high as 51.5 and 48.9%, respectively. A specific proportion of hydrophobic residues can effectively promote the interaction between antimicrobial peptides and bacterial cell membranes, and enhance their antimicrobial efficacy (Hancock and Sahl, 2006).

Phylogenetic analysis revealed that Han-Def1 and Han-Def2 are nested within a prominent evolutionary clade of Ixodidae defensins, clustering with sequences from several ixodid genera, including *Rhipicephalus*. This is consistent with the species taxonomic relationship and also implies the structural and functional conservation of Ixodidae defensins (Chrudimská et al., 2011; Wang et al., 2015). However, Ixodidae defensins did not form a single evolutionary clade, but were scattered in one major clade and three smaller clades. This observation is consistent with the conserved motif analysis. Among the six conserved motifs identified by the MEME platform, Motifs 1–2–3-4-6 were determined to be specific to Ixodidae defensins. Those members that did not cluster with the main Ixodidae clade lacked one or more key conserved motifs specific to this family. The absence of such conserved structural units may lead to subtle variations in their three-dimensional structure or surface properties, resulting in separation from the main Ixodidae clade in phylogenetic relationships. This reflects that during the evolution of Ixodidae defensins, while maintaining the core CSαβ fold, their sequence and motif composition have also undergone significant differentiation, which may provide a basis for their functional diversity and adaptation to different pathogen selection pressures (Cabezas-Cruz et al., 2019; Schmitt et al., 2016).

Functional experiments demonstrated that rDef1 and rDef2 possess potent bactericidal activity against Gram-positive bacteria *S. aureus*, *E. faecalis*, and *B. subtilis* but lack efficacy against *P. aeruginosa* and *E. coli*. This antimicrobial spectrum is consistent with several tick defensins showing similar activity profiles, such as persulcatusin from *Ixodes persulcatus* and HEdefensin from *Haemaphysalis longicornis*, which mainly exhibit activity against Gram-positive bacteria, particularly *S. aureus and M. luteus* (Miyoshi et al., 2017; Yada et al., 2018). In contrast, other tick defensins display broader antimicrobial spectra that include Gram-negative bacteria. For example, HlDFS1 and HlDFS2 from *H. longicornis* showed broad-spectrum activity against both Gram-positive and Gram-negative bacteria, including *E. coli* and *Borrelia burgdorferi*, and were also reported to inhibit antibiotic-resistant *A. baumannii* (Sun et al., 2017). In addition, holosins 2 and 3 from *Ixodes holocyclus* were reported to exhibit wide antimicrobial activity, with holosin 2 and its *γ*-core showing activity against the Gram-negative bacterium *E. coli* (Cabezas-Cruz et al., 2019).

These comparisons suggest that the difference between Han-Def1/2 and broader-spectrum tick defensins may not depend solely on overall cationicity, but also on the topology of positively charged residues and the amphipathic organization of the peptide surface. While Han-Def1 and Han-Def2 are strongly cationic (pI 9.38 and 9.99, respectively), broader-spectrum defensins may possess more favorable local charge clustering or amphipathic patterns that facilitate interaction with and destabilization of the lipopolysaccharide (LPS) layer of Gram-negative bacteria (Schmitt et al., 2016). The inability of Han-Def1 and Han-Def2 to overcome the tightly packed LPS barrier suggests that their surface properties are optimized for interacting with the teichoic-acid-rich envelope of Gram-positive species (Leannec-Rialland et al., 2021). However, as this trend is observed across a limited number of representative strains, further screening against a broader repertoire of Gram-negative pathogens is warranted to accurately define the functional boundaries of their antimicrobial spectrum.

Interestingly, our data reveal a subtle functional specialization between these two isoforms that further characterizes their biological roles. While rDef1 exhibited overall superior antibacterial potency, rDef2 demonstrated a more favorable safety profile and slightly enhanced antioxidant capacity. Specifically, at 750 μg/mL (182 μM for rDef1 and 175 μM for rDef2), rDef2 caused a lower hemolysis rate (1.31%) compared to rDef1 (1.51%), both of which remain well below the 2% threshold typically reported for safe tick defensins (Chrudimská et al., 2011). Furthermore, rDef2 showed higher free radical scavenging activity (92.75 μg Trolox/mL) than rDef1 (82.83 μg Trolox/mL). This high discriminative capacity between prokaryotic and eukaryotic membranes, combined with dual-function properties, suggests that minor sequence variations allow these defensins to provide a balanced immune response: rDef1 may prioritize rapid pathogen clearance, while rDef2 contributes more to maintaining redox homeostasis.

The biological significance of this dual-functionality, characterized by simultaneous antimicrobial and antioxidant activities, is closely tied to tick physiology. The identification of N-terminal signal peptides points toward Han-Def1 and Han-Def2 being processed through the classical secretory pathway, suggesting they function as components of the tick’s extracellular immune arsenal. In hard ticks, such defensins are typically synthesized in the fat body and subsequently released into the hemocoel to provide systemic protection against microbes that breach the midgut barrier, or expressed in the salivary glands for secretion into the tick-host interface (Sonenshine and Macaluso, 2017). During blood-feeding, ticks face a dual challenge: the threat of invading pathogens and the surge of reactive oxygen species (ROS) generated from heme degradation and the host’s inflammatory response (Hernandez et al., 2022). By scavenging excess free radicals, these defensins help regulate the redox balance of the local microenvironment. This strategy not only neutralizes microbes but also protects the tick’s own tissues from oxidative damage, thereby facilitating successful parasitism (Sonenshine and Macaluso, 2017). Future studies using TEM or SEM, together with membrane permeability assays, would help clarify whether these structural differences are directly associated with distinct membrane-disruptive behaviors.

## 5 Conclusion

In this study, two novel defensins derived from *H. anatolicum* were successfully identified and characterized. These peptides possess a typical cationic cysteine-stabilized *α*/*β* (CSαβ) structure and conserved molecular features characteristic of Ixodidae defensins. Functional experiments demonstrated that Han-Def1 and Han-Def2 exhibit antimicrobial activity against Gram-positive bacteria, complemented by significant *in vitro* antioxidant capacity. Furthermore, their minimal hemolytic activity suggests low toxicity toward mammalian erythrocytes. The dual antimicrobial and antioxidant properties, combined with their structural stability, make these defensins promising candidate molecules for combating drug-resistant Gram-positive bacterial infections and managing oxidative stress at the tick-host interface.

## Data Availability

The datasets presented in this study can be found in online repositories. The names of the repository/repositories and accession number(s) can be found in the article/Supplementary material.

## Footnotes

1
http://www.ncbi.nlm.nih.gov/geo/

2
https://meme-suite.org/meme/tools/meme

3
https://www.ncbi.nlm.nih.gov/Structure/cdd/wrpsb.cgi

4
https://web.expasy.org/protparam/protparam-doc.html

5
https://web.expasy.org/protscale/

6
https://services.healthtech.dtu.dk/services/SignalP-6.0/

7
https://services.healthtech.dtu.dk/services/TMHMM-2.0/

8
https://prosite.expasy.org/

9
https://www.novopro.cn/tools/secondary-structure-prediction.html

10
https://alphafoldserver.com/

11
https://iupred3.elte.hu
