# Supplementary material for: Characterization of two novel defensins Han-Def1 and Han-Def2 from Hyalomma anatolicum
Source: Front Microbiol. 2026 May 14;17:1823759. doi: 10.3389/fmicb.2026.1823759 (PMC13216044; doi:10.3389/fmicb.2026.1823759)
Supplement: Supplementary file 1 [file Table_1.docx]

Supplementary Material

# 1 Supplementary Data

**Cloning and Double Enzyme Digestion Verification of Han-Def1 and Han-Def2 Genes**

To obtain biologically active defensin proteins, the mature peptide coding sequences of Han-Def1 and Han-Def2 genes were successfully cloned from the cDNA library of *H. anatolicum*. The target bands appeared at 147 bp (Figure S2). Sequencing results confirmed that the obtained sequences were completely consistent with the previous transcriptome data and bioinformatics predictions, with no mutations occurring. These sequences have been deposited in the GenBank database under accession numbers PX972500 (Han-Def1) and PX972501 (Han-Def2). Subsequently, The Han-Def1 and Han-Def2 genes were amplified by PCR and directionally inserted into the multiple cloning site of the prokaryotic expression vector pET-32a+ using *EcoR*I and *Xho*I restriction sites, respectively. An enterokinase cleavage sequence was inserted at the N-terminus of each sequence to construct recombinant expression plasmids pET-32a-EK-rDef1 and pET-32a-EK-rDef2. Sequencing results showed that the inserted sequences were correct, indicating the successful construction of the recombinant plasmids (Figure S3 and Figure S4).

# 2 Supplementary Figures and Tables

## 2.1 Supplementary Figures


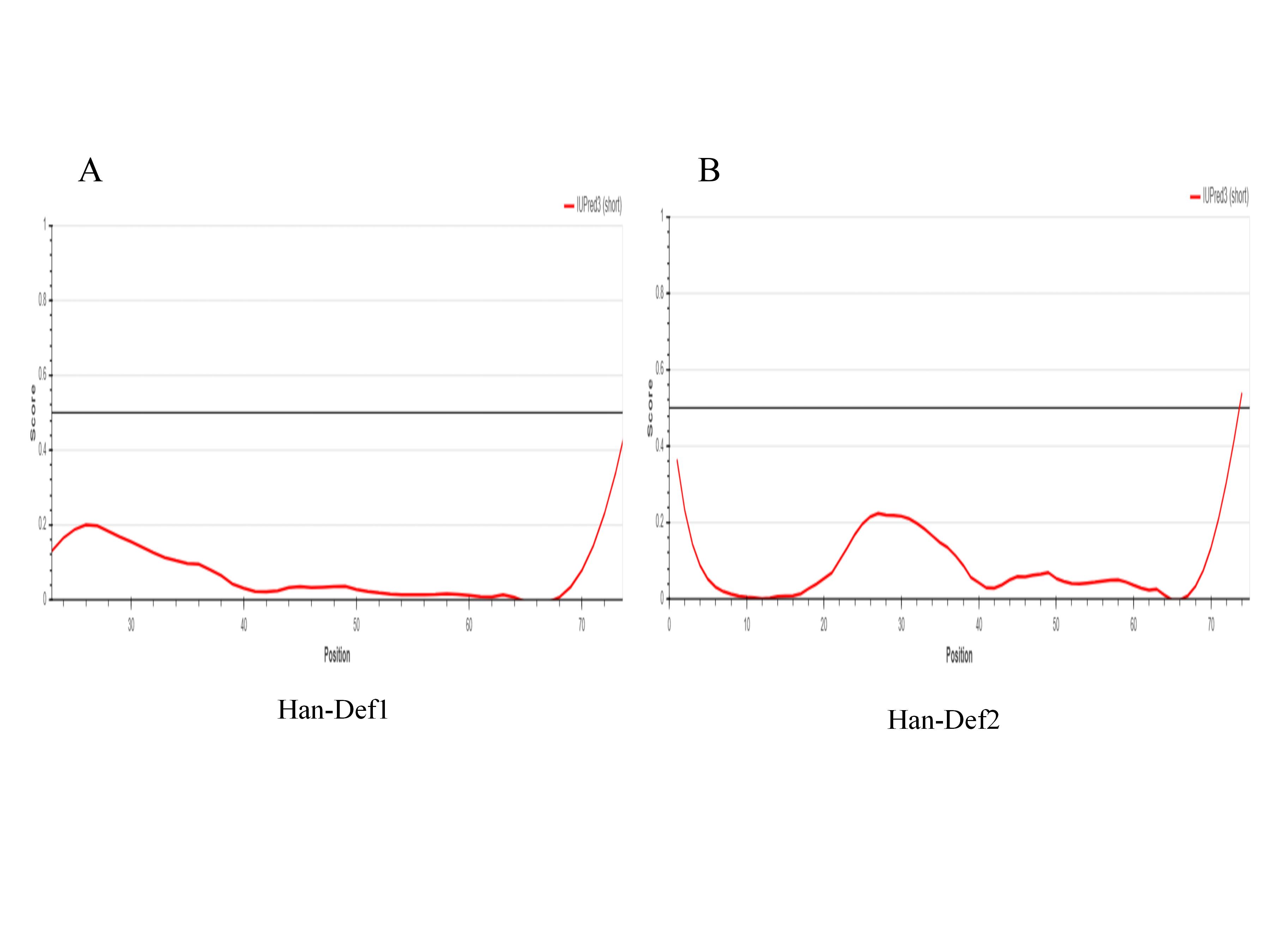


**Supplementary Figure 1.** Prediction of intrinsically disordered regions in Han-Def1 and Han-Def2 by IUPred3. Disorder tendency plots of Han-Def1 (A) and Han-Def2 (B) were generated using IUPred3 with the short disorder prediction mode. Residues with disorder scores > 0.5 were considered intrinsically disordered. Nearly all residues in both sequences scored below 0.5, with only the C-terminal residue of Han-Def2 exceeding the threshold, indicating the absence of extensive intrinsically disordered regions and supporting the compact folded structure of both defensins.

**Supplementary Figure 2.** PCR amplification results of the *Han-Def1* and *Han-Def2* genes. Lane M, DNA marker; lane 1, PCR product of Han-Def1; lane 3, PCR product of Han-Def2. The amplified fragments corresponded to the expected sizes of the target genes.


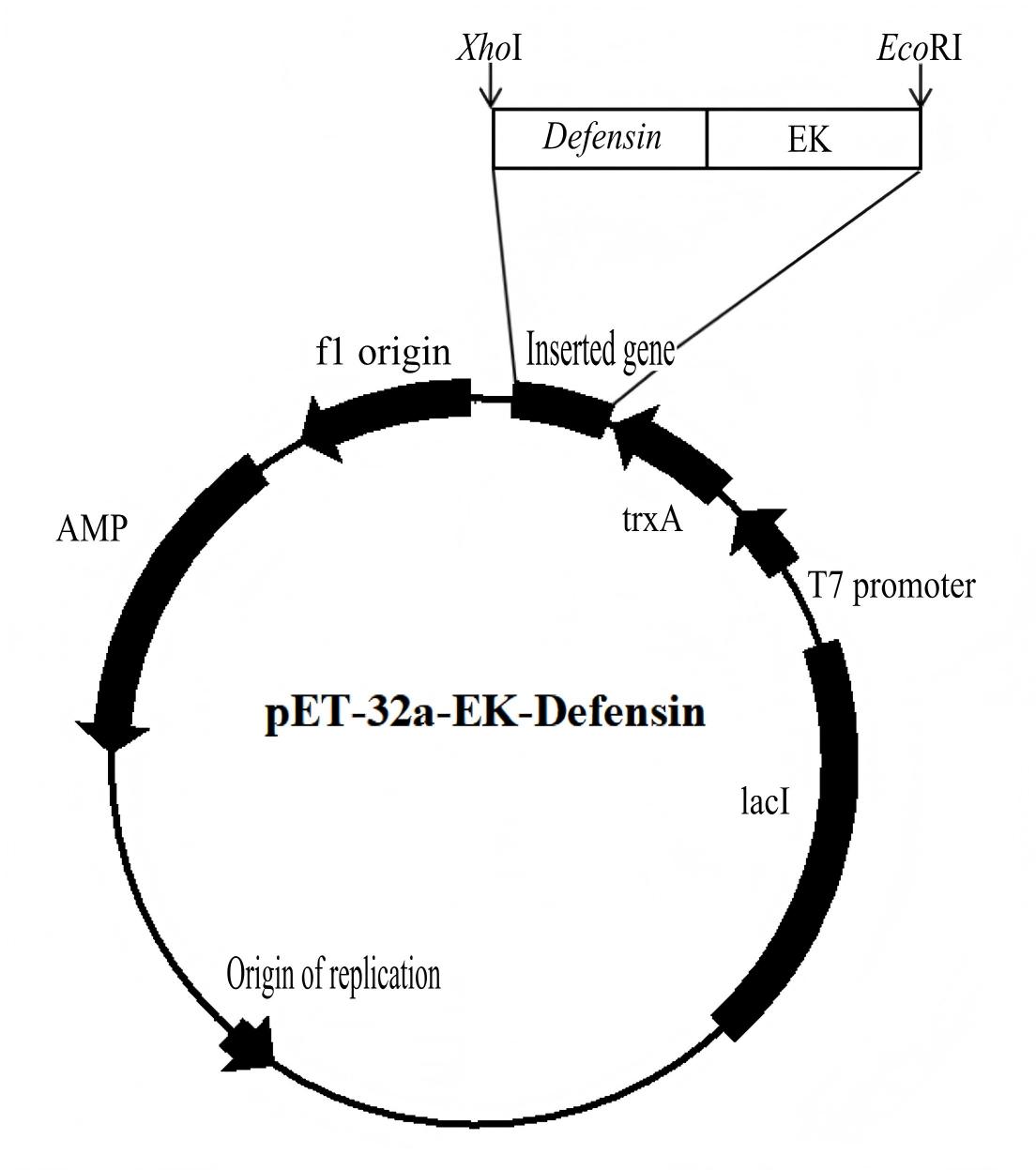


**Supplementary Figure 3.** Schematic diagram of the construction of expression vectors for rDef1 and rDef2. The mature peptide coding sequence of each defensin was inserted into the pET-32a^+^ vector between the *Eco*RI and *Xho*I restriction sites. An enterokinase (EK) cleavage site was introduced upstream of the defensin sequence to allow removal of the fusion tag after recombinant expression and purification.

**Supplementary Figure 4.** Verification of recombinant plasmid construction by double enzyme digestion. Recombinant plasmids for rDef1 and rDef2 were digested with *Eco*RI and *Xho*I to confirm successful insertion of the target fragments into the pET-32a vector. Left, digestion pattern of the rDef1 recombinant plasmid; right, digestion pattern of the rDef2 recombinant plasmid. The observed bands corresponded to the expected insert and vector backbone sizes.

## 2.2 Supplementary Tables

**Supplementary Table 1** The database of known defensins of ticks.

| **Tick species** | **NCBI accession number** | **Sequence information** |
| --- | --- | --- |
| *Hyalomma anatolicum* | PX972500 | MKVLAIALVFLLVAGLTSTTVAEAEESAVAHVRVRRGFGCPLNQGACHNHCRSIGRRGGYCAGIIKQTCTCYRN |
| *Hyalomma anatolicum* | PX972501 | MKVLTIALIVVLLTGLVSTAAAQDGEGDVAHVRVRRGFGCPFNQGACHRHCRSTRRRGGFCSGIIKQTCTCYRN |
| *Ornithodoros moubata* | BAB41027 | MNKLFIVALVVALAVATMAQEVHDDVEEQSVPRVRRGYGCPFNQYQCHSHCRGIRGYKGGYCTGRFKQTCKCY |
| *Ornithodoros moubata* | BAB41028 | MNKLFIVALVVALAVATMAQEVHNDVEEQSVPRVRRGYGCPFNQYQCHSHCSGIRGYKGGYCKGTFKQTCKCY |
| *Ornithodoros papillipes* | ACJ04425 | MNKLFIVALVVALAVATMAQEVHDDVEEQSVPRVRRGYGCPFNQYQCHSHCSGIRGYKGGYCKGTFKQTCKCY |
| *Ornithodoros papillipes* | ACJ04426 | MNKLFIVALVVALAVATMAQEVHDDVEEQSVPRVRRGYGCPFNQYQCHSHCRGIRGYKGGYCTGRFKQTCKCY |
| *Alectorobius puertoricensis* | ACJ04429 | MNKLFIVALVAALAVATMAQEVHNDVEEQSVPRVRRGYGCPFNQYQCHSHCSGIRGYKGGYCKGTFKQTCKCY |
| *Alectorobius puertoricensis* | ACJ04430 | MNKLFIVALVVALAVATMAQEVHDDVEEQSVPRVRRGYGCPFNQYQCHSHCRGIRGYKGGYCTGRFKQTC  KCY |
| *Ornithodoros rostratus* | ACJ04428 | MNKLFIVALVVAPAVATMAQEVHNDVEEQSVPRVRRGYGCPFNQYQCHSHCSGIRGYKGGYCKGTFKQTCKCY |
| *Ornithodoros tartakovskyi* | ACJ04431 | MNKLFIVALVVALAVATMAQEVHNDVEEQSVPRVRRGYGCPFNQYQCHSHCSGIRGYKGGYCKGTFKQTCKCY |
| *Ornithodoros turicata* | QIG55621 | MKTVFVIALVFALAVASMAQDVDDVEESSAVRVRRGYGCPFNQYQCHSHCSGIRGYKGGYCKGLFKQTCTCY |
| *Ornithodoros turicata* | QIG55622 | MKVLCFLLLLLLTGLLTSRAAVLDTRRDPEDGTGNDCPHNEIACTLKCERDGFAYGRCTGLVLDQKCECIA |
| *Amblyomma americanum* | ABI74752 | MKVLAVAFIFVLVAGLVSTADEEDKSQVPLVRVRRGFGCPFNQYQCHSHCLSIGRRGGYCGGSFKTTCTCYN |
| *Amblyomma hebraeum* | AAR97290 | MATVRNSRPEAAGEPSGVSSTEGDWRHIEKRDVSYQGEGNTRRFDNPFGCPADEGKCFDHCNNKAYDIGYCGGSYRATCVCYRK |
| *Amblyomma hebraeum* | AAR97291 | MATQRREISWTFGPLYTWRTTKGYGTTLETTNATSTSSKPSRRYENPYGCPTDEGKCFDRCNDSEFEGGYCGGSYRATCVCYRT |
| *Dermacentor marginatus* | ACJ04433 | MRGLCICLVFILVCGLLTATAAVPAESEAAHLRVRRGFGCPLNQGACHNHCRSIRRRGGYCSGIIKQTCTCYRN |
| *Dermacentor reticulatus* | ACJ04434 | MRGLCICLVFILVCGLLTATAAVPAESEAAHLRVRRGGYCSGIIKQTCTCYRN |
| *Dermacentor silvarum* | AJG42673 | MRGLCICLVFILVCGLLTATAAVPAESEAAHLRVRRGFGCPLNQGACHNHCRSIRRRGGYCSGIIKQTCTCYRN |
| *Dermacentor silvarum* | QJD21999 | MKFTSAIFLIAVLAAFLAMMTAAEESSENMGGAHSDRGGCPDADKCTKYCQKQGISVGKCAKPVNACVCIL |
| *Dermacentor variabilis* | AAO24323 | MRGLCICLVFLLVCGLVSATAAAPAESEVAHLRVRRGFGCPLNQGACHNHCRSIRRRGGYCSGIIKQTCTCYRN |
| *Dermacentor variabilis* | AAO18363 | MRFTSAIFLVAVIAAFVVMITATGERSEERSEEARASGCKADACKSYCKSLGSGGGYCDQGTWCVCN |
| *Haemaphysalis longicornis* | BAD93183 | MKVLAVALIFVLVAGLFCTAAAQDDESDVPHVRVRRGFGCPLNQGACHNHCRSIGRRGGYCAGIIKQTCTCYRK |
| *Haemaphysalis longicornis* | ABO28925 | MKVFSALFLVGLLLAFLAFAAGDEEDSSKPLVRVRRGFGCPFDERACHAHCQSVGRRGGYCGNFRMTCYCYKN |
| *Ixodes persulcatus* | BAH09304 | MRVVAVALIALLVAGAFMTSSAQEEENQVAHVRVRRGFGCPFNQGACHRHCRSIGRRGGYCAGLFKQTCTCYSR |
| *Ixodes ricinus* | AAP94724 | MKVLAVSLAFLLIAGLISTSLAQNEEGGEKELVRVRRGGYYCPFFQDKCHRHCRSFGRKAGYCGGFLKKTCICVMK |
| *Ixodes ricinus* | ABC88432 | MKVLAVSLAFLLIAGLISTSLAENDEGGEKELVRVRRGGYYCPFRQDKCHRHCRSFGRKAGYCGGFLKKTCICV |
| *Ixodes scapularis* | EEC08934 | MKAVAIALVVMMIAGLISTSCSQEDDSQVAHVRVRRGFGCPLNQGACHNHCRSIKRRGGYCSGIIKQTCTCYRK |
| *Ixodes scapularis* | EEC13914 | MKVVGIALVVRLFSFSCSQGVHSQVPHVRVRRAFGCPFDQGTCHSHCRSIRRRGERCSGFAKRTCTCYQK |
| *Rhipicephalus microplus* | AAO48943 | MRGIYICLXFVLXCGLVSGLADVPAESEMAHLRVRRGFGCPFNQGACHRHCRSIRRRGGYCAGLIKQTCTCYRN |
| *Argas monolakensis* | ABI52817 | MNKSLVIVLVLAIAVAATTAQSVDEPERSHGRVRRGYGCPFNQYECHNHCKGVPGYKGGYCDGFLKMTCRCY |
| *Haemaphysalis longicornis* | B2MW54 | MAESTTTCFLLLVTGYVTAVMSEEAHLRSRRDFGCGQGMIFMCQRRCMRLYPGSTGFCRGFRCMCDTHIPLRPPFMVG |

**Supplementary Table 2. The PCR Primer Sequences and Reaction System for the *Han-def1/Han-def2* Genes of *Hyalomma anatolicum***

| **Primer pairs** | **(5’ to 3’ end)** | **Anotation** | **PCR Product** | **Reaction condition** | **Amplification system** |
| --- | --- | --- | --- | --- | --- |
| Han-def1-F  Han-def1-R | GGAATTCGACGACGACGACAAGGGCTTTGGGTGCCCCTTTA  CCCTCGAGCTAATTACGGTAACAGGTGC | Defensin | 147 | Thermal cycling conditions included an initial denaturation step of 95°C for 4 min, followed by 35 cycles of 95°C for 30 s, 66.9°C for 30 s, and 72°C for 45 s, with a final extension step of 72 °C for 5 min. | 50μL reaction, 2×PCR Mix 25μL, 1μL each for forward and reverse primers, 2μL of sample DNA, 22μL of ddH_2_O. |
| Han-def2-F  Han-def2-R | GGAATTCGACGACGACGACAAGGGTTTTGGTTGTCCTCTT  CCCTCGAGCTAGTTGCGGTAGCAAGT | Defensin | 147 | Thermal cycling conditions included an initial denaturation step of 95°C for 4 min, followed by 35 cycles of 95°C for 30 s, 67.9°C for 30 s, and 72°C for 45 s, with a final extension step of 72 °C for 5 min. |  |
